# Temperature gradient analyzers for compact high-resolution X-ray spectrometers

**DOI:** 10.1107/S0909049509043167

**Published:** 2009-11-26

**Authors:** D. Ishikawa, A. Q. R. Baron

**Affiliations:** a Materials Dynamics Laboratory, RIKEN/SPring-8 Center, 1-1-1 Kouto, Sayo-cho, Sayo-gun, Hyogo 679-5148, Japan; b Research and Utilization Division, JASRI/SPring-8, 1-1-1 Kouto, Sayo-cho, Sayo-gun, Hyogo 679-5198, Japan

**Keywords:** X-ray spectrometers, analyzer crystals, inelastic X-ray scattering, atomic dynamics, electronic dynamics

## Abstract

Compact high-resolution X-ray spectrometers with a one-dimensional temperature gradient at the analyzer crystal are considered. This gradient, combined with the use of a position-sensitive detector, makes it possible to relax the usual Rowland-circle condition, allowing increased space at the sample position for a given energy resolution or arm radius. Thus, for example, it is estimated that ∼meV resolution is possible with a 3 m analyzer arm and 200 mm clearance between the sample and detector. Simple analytic formulae are provided, supported by excellent agreement with ray-tracing simulations. One variation of this method also allows the detector position sensitivity to be used to determine momentum transfer, effectively improving momentum resolution without reducing (slitting down) the analyzer size. Application to medium-resolution (∼10–100 meV) inelastic X-ray scattering spectrometers with large angular acceptance is discussed, where this method also allows increased space at the sample. In some cases the application of a temperature gradient can improve the energy resolution even with a single-element detector.

## Abstract 2

A temperature gradient applied to analyzer crystals allows relaxation of the Roland-circle geometry for meV-resolution spectrometers.

## 1. Introduction   

Non-resonant inelastic X-ray scattering (IXS), with a resolution of less than ∼100 meV, is a rapidly growing field. In the high (meV) resolution limit, one has access to atomic dynamics, which are important in many phase transitions, and especially in the context of modern materials science, where the phonons are a crucial component of correlated systems. Atomic dynamics are also intimately connected with the behavior and structure of disordered materials such as liquids and glasses. Medium-resolution spectrometers, with higher intensity from relaxed resolution, can be used to measure electronic dynamics, with direct access to band structure, the multi-polarity of the electronic transitions, and to possible correlations between electronic transitions (*e.g.* dispersing excitations such as orbitons). The combination of improved instrumentation and increased access to sophisticated calculations makes measurement of the dynamic structure factor for both atoms and electrons an increasingly attractive endeavor, especially if high resolution can be obtained.

There are presently many efforts under way to improve the present generation of spectrometers, and to design the next generation of instruments, especially with new third-generation sources coming on line. In this context, the relatively recent suggestion of ‘dispersion compensation’ by Huotari and co-workers (Huotari *et al.*, 2005 ▶), allowing improved resolution with a fixed-size spectrometer, or a smaller spectrometer for a fixed resolution, is of great interest. In principle, this is particularly true for high-resolution (∼meV) spectrometers (Dorner & Peisl, 1983 ▶; Sette *et al.*, 1998 ▶; Burkel, 1991 ▶; Baron *et al.*, 2000 ▶; Sinn *et al.*, 2001 ▶) where the size of the 2θ (analyzer) arms can be ∼10 m, which is very large given the limited space on the experimental floor of synchrotron radiation facilities. However, the work of Huotari *et al.* focused primarily on medium (20–100 meV) resolution, and is difficult to extend to ∼meV resolution because clearance between the sample and the detector becomes extremely restrictive. In the scheme suggested by Huotari *et al.*, this clearance, *d*, scales as *d* = 4∊*R* ^2^/*p* where *R* is the arm radius, ∊ = Δ*E*/*E* is the fractional energy resolution, and *p* is the detector pixel size. Thus, for example, taking *R* = 3 m, *p* = 0.1 mm, Δ*E* = 0.3 meV at *E* = 26 keV gives *d* = 4.2 mm, which severely limits the space for sample environment (one would really like ∼100 mm clearance, or more).

The present paper discusses how to achieve ∼meV resolution with a short analyzer arm, while retaining a relatively large (200 mm) clearance between the detector and the sample. We show that the application of a one-dimensional temperature gradient to the usual analyzer crystals, resulting in a corresponding gradient in the lattice spacing, allows relaxation of the Rowland-circle condition while retaining high resolution. We present a detailed analytical treatment of the various contributions supported by excellent agreement with ray-tracing simulations. While focused primarily on ∼meV energy resolution and ∼10 mrad angular acceptance (high resolution), we also consider ∼10 meV resolution and ∼100 mrad acceptance (medium resolution).

The article is organized as follows. §2 reviews the basic concepts, introduces the limit of applying dispersion compensation for high-resolution work, and, qualitatively, introduces the analyzer temperature gradient. §3 presents a detailed quantitative analytic treatment of two different types of temperature gradient set-ups, and §4 discusses ray-tracing simulations and includes the effects of imperfect analyzer figure. The results for meV analyzers are discussed in §5 and application to medium resolution is covered in §6. Practical aspects, including detector size, momentum resolution and backgrounds are discussed in §7. Test results for one possible temperature gradient scheme are given in §8 and conclusions are presented in §9.

## 2. Basic concepts   

### 2.1. Crystal optics   

At present, sub-eV-resolution X-ray spectrometers generally use crystal analyzers; the energy resolution of most detectors remains ∼100 eV in the hard X-ray region and, while bolometers can achieve ∼eV resolution for softer X-rays, they are far from the 0.1 eV level. Thus crystal analyzers are almost^1^ the only option. Typical resolutions are given in Table 1 ▶. However, for crystal analyzers, one is severely limited by the angular acceptance of Bragg reflections in the perfect crystals, which is typically of the order of microradians, while to obtain reasonable count rates one typically desires large angular acceptance, *e.g.* 1 to 100 mrad, depending on the details of the experiment. The relation between angular acceptance and energy resolution for diffraction from a flat perfect crystal is derived from Bragg’s law as 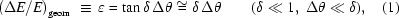
where *E* is the photon energy, δ ≃ π/2 − θ_B_ (θ_B_ is the Bragg angle) is a deviation angle from exact backscattering of the crystal, and Δ*E* is the geometric contribution to the energy resolution owing to a divergence of Δθ. Given, for example, a desired^2^ upper limit of a geometric contribution to the resolution of 0.3 meV at 26 keV and a typical operating angle of δ ≃ 0.2 mrad one finds the angular acceptance of a flat crystal is only Δθ < ∼60 µrad.

**Figure 1:**
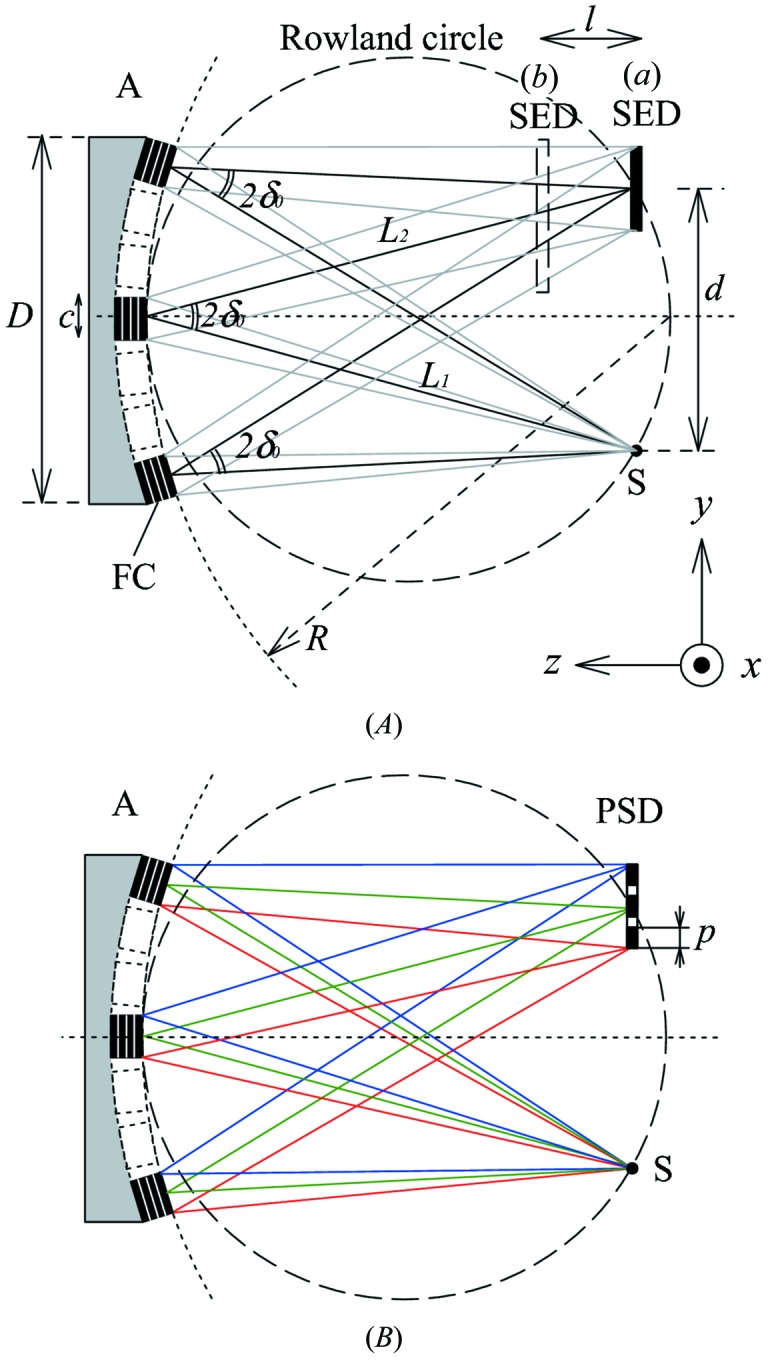
Schematic of conventional IXS analyzer geometries using diced crystal analyzers. (*A*) Rowland circle with a single detector either on the focus (solid) or offset (broken). (*B*) The conventional ‘dispersion compensation’ set-up with a position-sensitive detector on the Rowland circle. S: sample; A: analyzer; SED: single-element detector; PSD: position-sensitive detector; FC: flat crystallite. Parameters are listed in Table 2 ▶. The figure dimensions are exaggerated for clarity (*L*_1_, *L*_2_ 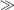 *D*, *l* 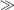 *d*, *c*).

To move beyond this severe limit, one usually creates a figured analyzer operating in the Rowland circle condition, where the shape of the analyzer crystal is chosen so that all rays from a point source hit it at a fixed angle, reducing or removing the geometric contribution from equation (1). For the highest resolution, one uses diced analyzers to remove strain from bending a crystal (Fig. 1*A* ▶). The angular limit is then set by the crystallite size of the analyzer crystals [see discussions by Masciovecchio *et al.* (1996*a* ▶,*b* ▶)]. In this geometry the crystallite size in the diffraction plane, *c*, sets the angular scale Δθ ≃ *c*/*L*_1_ (*L*_1_ is the sample-to-analyzer distance) giving a contribution to the energy resolution (Fig. 1*A* ▶),
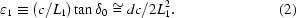
The second approximation is the first-order term assuming the detector is offset a distance *d* from the sample. The cube size, owing to issues of fabrication, is usually ∼1 mm. One then finds that a 0.3 meV geometric contribution at 26 keV for a 10 m arm allows *d* ≃ 2.3 mm. As *L*_1_ (the arm radius) is reduced, this quickly becomes an even more severe limit, with *d* scaling as 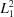.

### 2.2. Dispersion compensation   

Huotari and co-workers (Huotari *et al.*, 2005 ▶) introduced the use of a position-sensitive detector in the focal plane, essentially combining a focusing analyzer with a dispersive detector (see Fig. 1*B* ▶). They showed that, assuming a sufficiently perfect analyzer figure, the block size of the crystal analyzer in (2) could be replaced by the pixel size, *p*, of the detector,
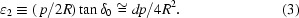
However, this relies on strict observance of the Rowland-circle condition, with the detector directly above the sample (*L*_1_ = *L*_2_ = *R*). For high resolution, ∼meV, this is a very severe constraint that limits the available space at the sample to a few millimeters. For example, the detector–sample clearance, assuming a contribution of 0.3 meV at 26 keV (∊ = 1 × 10^−8^) when *R* = 5 m and *p* = 0.1 mm is *d* ≃ 10 mm. This improves on the previous 2.3 mm of §2.1 but any sort of sample environment (refrigerators, furnaces, high-pressure cells) remains problematic.

### 2.3. Demagnification contribution and failure of dispersion compensation   

**Figure 2:**
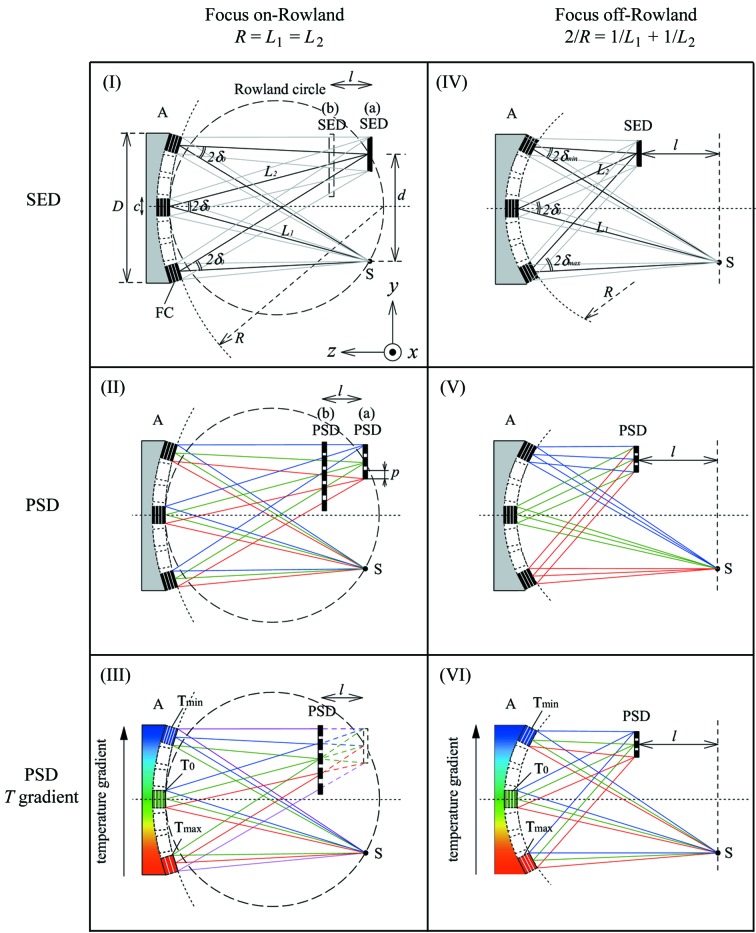
Schematic of IXS analyzer geometries using diced crystal analyzers. (I) Positioned along a vertical Rowland circle with single-element detector (including detector offset with focusing on-Rowland: broken square). (II) (*a*) Rowland geometry with a PSD, (*b*) focal point on the Rowland circle but detector off the circle. (III) Focusing on-Rowland but detector in front of focal point. (IV) Focusing off-Rowland with a single-element detector, demagnification contribution causes chromatic aberration. (V) Detector offset with PSD; energy–position correspondence in (II) is broken in (V). (VI) Temperature gradient of the analyzer crystal (*T*_min_ < *T*_0_ < *T*_max_); temperature correction reduces chromatic aberration and allows off-Rowland geometry to be used. Colored lines indicate dispersed energy for clarity, *e.g.* the different color of the rays are focused on the center of the detector in case (V), while one color of the rays are focused on the same position in case (VI).

One can consider focusing off the Rowland circle to make space around the sample [Fig. 2(V) ▶]. However, this introduces variation in the Bragg angle over the analyzer surface leading to what has been called a demagnification contribution (Burkel, 1991 ▶) to the resolution given by 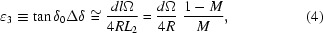
where Δδ is the distribution of angles onto the analyzer defined as Δδ ≡ (δ_max_ − δ_min_); here δ_max_ and δ_min_ are maximum and minimum δ value shown [see also Figs. 2(I)(*b*), 2(IV), 2(V) ▶ and Table 2 ▶], and Ω is the angle of scattered rays intercepted by the analyzer, Ω ≡ *D*/*L*_1_, *D* is the analyzer size, and *M* = *L*_2_/*L*_1_ (see Table 2 ▶). Choosing, for example, *d* = 3 mm, *L*_1_ = 5 m (δ_0_ ≃ 0.3 mrad), *l* = 200 mm and Ω = 10 mrad, one finds a geometric contribution of ∊_3_ ≃ 6.4 × 10^−8^ or Δ*E* = 1.6 meV at 26 keV. This significantly limits the achievable energy resolution.

### 2.4. Temperature gradient analyzers: qualitative   

To a first approximation, the temperature gradient we suggest here may be considered as a way of modifying the lattice constant to compensate for the demagnification contribution, essentially varying the *d*-spacing to correct for the variation in the Bragg angle, δ, over the analyzer. This allows us to introduce the idea, and sets the scale for the required gradient, though a different, and, in some cases, better, method will also be described below. The magnitude of the required temperature gradient over the analyzer is roughly given as Δ*T* = ∊_3_/α where α is the thermal expansion coefficient of the analyzer and ∊_3_ is the demagnification contribution from equation (4). Taking the previous case (*d* = 3 mm, *L*_1_ = 5 m, 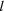 = 200 mm and Ω = 10 mrad), one can estimate the required gradient to be about 25 mK over a silicon analyzer operated at room temperature (α = 2.6 × 10^−6^ K^−1^). This is a small, but crucial, adjustment to achieving high resolution. It becomes more important as the arm radius is further reduced.

## 3. Temperature gradient analyzers: quantitative   

Detailed discussion of the temperature gradient depends on the precise focusing conditions. In the preceding section, the temperature gradient was introduced as a response to the demagnification contribution when one moved the analyzer focus off the Rowland circle. However, there are actually two limiting cases: one where the analyzer focus remains on the Rowland circle and only the detector is moved away from the sample, and one where both the analyzer focus and the detector are moved off the Rowland circle together. These will be referred to as the ‘on-Rowland’ and ‘off-Rowland’ cases, respectively, where the designation refers to the position of the analyzer focus. These are shown in Fig. 2 ▶, where cases (I)–(III) are all on-Rowland while (IV)–(VI) are off-Rowland. The temperature gradient can be used to improve the resolution in both cases. Considering resolution only, the on-Rowland case is better. However, practical considerations (beam size and detector noise) can make the off-Rowland geometry attractive.

Before proceeding, we introduce another important parameter, the clearance between the divergent beam scattered from the sample to the analyzer and the beam reflected from the analyzer into the detector. The minimum clearance, so that the detector does not occlude the analyzers and so that the entire reflected beam is collected by the analyzer, is denoted *d*_min_. Note that choosing *d* = *d*_min_ leaves no space for either a border around the detector or for shielding. By default, we will take *d* = *d*_min_ + 2 mm to allow for these.

### 3.1. Temperature gradient for focus on-Rowland [case (III)]   

**Figure 3:**
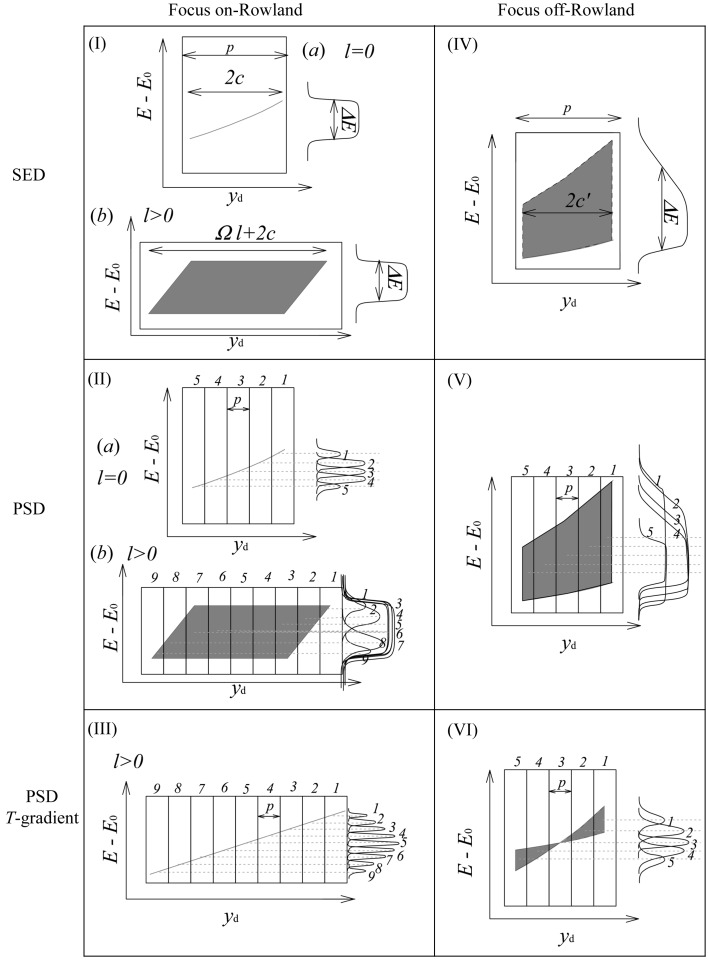
Schematic of the energy–position correlation in the six different geometries of Fig. 2 ▶. Right-hand line shapes are projections of the resolution function of each detector element. *E* − *E*_0_: relative energy, *y*_d_: detector *y*-position; *p*: detector pixel size; 2*c*: image size of the small cube crystal. 2*c*′: demagnified image size. The temperature gradient of the analyzer reduces the aberration [(III), (VI)].

Here we discuss the situation described by Fig. 2 ▶(III). The analyzer focus remains on the Rowland circle, so very near to the sample, but the detector is moved towards the analyzer to make space at the sample position. Applying a proper temperature gradient allows preservation of a (nearly) unique energy–position correlation in the detector despite the detector being out of the analyzer focus. Considering Fig. 3 ▶, the temperature gradient preserves the linear relationship between energy and position [shown in Fig. 3(II)(*a*) ▶], but increases its range [Fig. 3(III) ▶].

The exact form of the correlation between temperature and position on the analyzer is derived as follows. For a fixed angle of incidence the energy difference between rays reflected by two different crystal cubes having temperature *T* and *T*_0_ is Δ*E*/*E* = *d*_*hkl*_(*T*_0_)/*d*_*hkl*_(*T*) − 1, where *d*_*hkl*_(*T*) is the *d*-spacing at temperature *T*. Meanwhile, neglecting the cube size of the analyzer (*c* → 0) and using equation (3), the energy offset and detector vertical displacement, *y*_d_, are related by Δ*E*/*E* = (*y*_d_/2*R*′)tanδ_0_. Here, *R*′ satisfies 2/*R*′ = 1/*L*_1_ + 1/(*L*_1_ − l) and *y*_d_ can be replaced by the analyzer *y*-position (*y*_a_) in Fig. 2(VI) ▶ using *y*_d_ ≃ *l* *y*_a_/*L*_1_. Then the relation between *y*_a_ and temperature deviation Δ*T* (≡ *T* − *T*_0_) is 
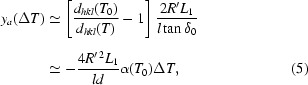
where
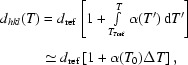
and the second equality assumes the thermal expansion coefficient, α(*T*), is approximately temperature independent. Precise values of α(*T*) for silicon may be found by Watanabe *et al.* (2004 ▶) and Okada & Tokumaru (1984 ▶), and a reference lattice constant *a*_ref_ = 5.43102 Å at *T*_ref_ = 295.65 K (Mohr & Taylor, 2000 ▶). Taking the center of the analyzer to be at temperature *T*_0_ = 300.000 K, we may write *d*_*hkl*_(*T*_0_)/*d*_*hkl*_(*T*) − 1 ≃ −α(*T*_0_) ≃ −2.627879 × 10^−9^Δ*T* [mK] + *O*(Δ*T*)^2^.

**Figure 4:**
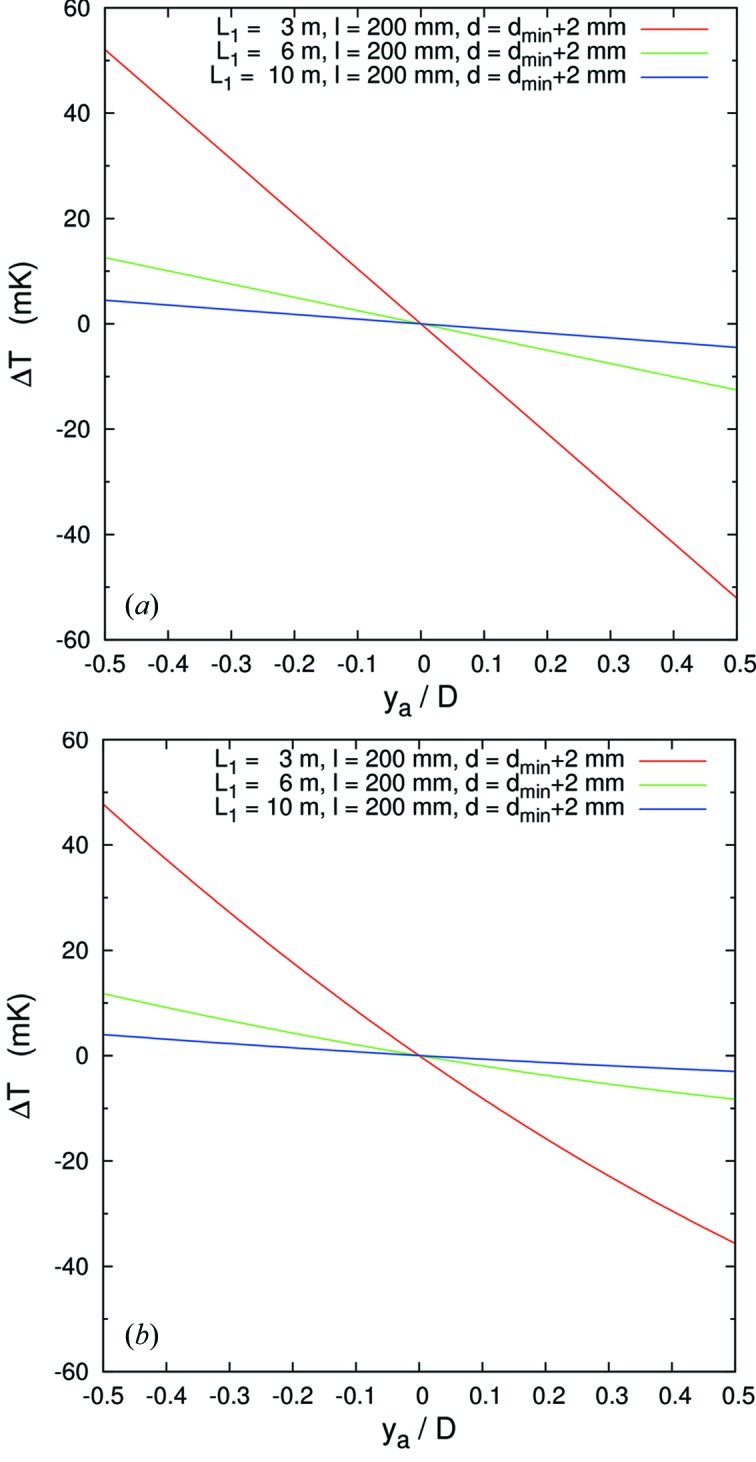
Temperature gradient curve Δ*T* as a function of analyzer *y*-position (*y*_a_) normalized by the analyzer dimension. Optical geometry is (*a*) focus on-Rowland [case (III)] and (*b*) focus off-Rowland [case (VI)]. Each geometry compares *L*_1_ = 3.0, 6.0 and 10.0 m and *D* = 30, 60, 100 mm. Note the more nearly linear behavior in (*a*).

Fig. 4(*a*) ▶ shows the required temperature gradient as a function of normalized analyzer dimension for *L*_1_ = 3, 6, 10 m, *l* = 200 mm and, as mentioned above, *d* = *d*_min_ + 2 mm. In this geometry, assuming perfect analyzer figure and a point source, *d*_min_ is given by 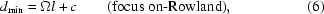
where Ω*l* is the vertical size of the beam to the analyzer at a distance *l* from the sample. The temperature gradient is linear and the ranges are ±52, ±12.6 and ±4.5 mK relative to the center of the analyzer, respectively.

### 3.2. Temperature gradient for focus off-Rowland [case (VI)]   

Here we discuss the situation described by case (VI) of Figs. 2 ▶ and 3 ▶. The analyzer focus remains in the detector as it is moved off the Rowland circle, introducing a demagnification contribution, which is then compensated by the temperature gradient. Considering Fig. 3 ▶, one can consider the gradient as a way of collapsing the dispersion over the detector [Fig. 3(V) ▶] to a more almost linear form [Fig. 3(VI) ▶]. This is essentially a first-order correction to the demagnification contribution. However, owing to the range of Bragg angles now going to the analyzer focal point, the slope of the energy dispersion *versus* detector position depends on the position in the analyzer where the reflection occurs, thus the correction is only perfect for one position in the detector. However, it still reduces the measured bandwidth. This may be analyzed in detail as follows. For an off-circle focus the analyzer radius, *R*, is given by the usual lens equation^3^

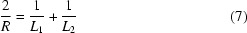
where, as shown in Fig. 2(IV) ▶ and Table 2 ▶, *L*_1_ is the distance from the sample to the analyzer, and *L*_2_ is that from the analyzer to the focal point. The detector is at *l* = *L*_1_ − *L*_2_. The required condition to keep the energy constant over the analyzer then becomes *d*_*hkl*_(*T*)cosδ = constant. Taking *T*_0_ and δ_0_ as the temperature and angle at the center of the analyzer, *y*_a_(Δ*T*) is expressed as 
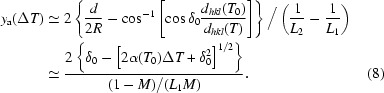
This may be inverted to give
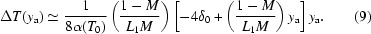
Note that, in contrast to §3.1, the second-order term is no longer negligible. Then the minimum detector offset in this geometry is given as 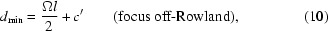
where the image from a single block of the analyzer will have a size reduced by the shorter path length to the detector, 2*c*′ = *c*(1 + *M*).

Fig. 4(*b*) ▶ shows the temperature gradient Δ*T* as a function of normalized analyzer dimension for parameters *L*_1_ = 3, 6 and 10 m and *l* = 200 mm and *d* = *d*_min_ + 2 mm. The temperature gradient is not linear, and ranges from +48 to −35, +11 to −9 and +4 to −3 mK, respectively. The energy–position correlation becomes quadratic as seen in Fig. 3(VI) ▶. The energy–position density is also not uniform and may yield asymmetric line shapes for the resolution function.

**Figure 5:**
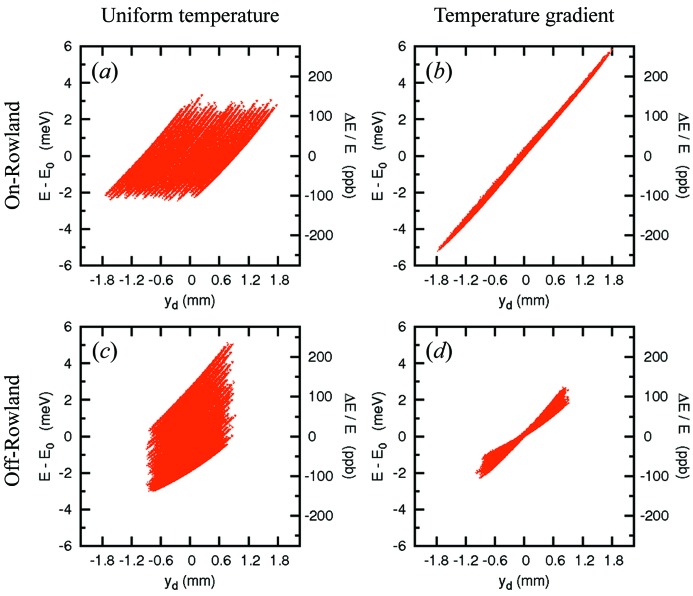
Energy–position correlation in the detector. (*a*) and (*b*) are for the on-Rowland case without or with temperature gradient of the analyzer crystal; (*c*) and (*d*) are for the off-Rowland geometry without or with temperature gradient. *E* = 21.747 keV for Si(11 11 11) backreflection case from the ray-tracing results. Optical geometry: *L*_1_ = 3 m, *l* = 200 mm and (*a*) *d* = 4.60 mm and (*b*) *d* = 3.56 mm are considered here. Contributions from analyzer slope error, σ_(*x*,*y*)_ = 20 µrad × 20 µrad, and source size σ_ss(*x*,*y*)_ = 20 µm × 20 µm, are included.

If the temperature gradient given by (8) is applied, then the full width of the energy distribution at the edge of the detector [seen in Fig. 5(*d*) ▶ or Fig. 9(*d*)] is ∊ = (*c*′/2*R*)Δδ. Assuming the detector pixel size is relatively small compared with the beam size, this contribution is reduced when the integration (with appropriate energy shift) over the detector is performed. In addition, we note that the quadratic dependence of the energy shift on the position in the analyzer leads to a concentration of the intensity near the central (small slope) line in these figures. Thus the practical contribution to the energy shift in this case is about (*c*′/2*R*)Δδ/4. This is, perhaps, more easily seen in Figs. 5(*c*) and 9(*c*), which, after applying the temperature gradient, are essentially compressed into Figs. 5(*d*) and 9(*d*), but the weighting remains very asymmetric. The pixel size contribution is most accurately represented by using δ_max_ in (3). The energy resolution in this case is then given by 
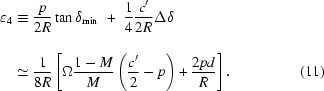
It is worth noting that the non-linear energy–position correlation of this geometry owing to the variation in δ over the analyzer surface leads to a slightly worse energy resolution. Also a non-linear temperature gradient may be difficult to achieve practically. However, in contrast to the focus on-Rowland case, the image size at the detector is reduced, as shown in Table 3 ▶. Therefore detector size can be smaller, reducing *d*_min_ and the detector background (see §7).

It is also worth noting that spherical aberration originates from the deviation from ideal aspherical shape (ellipsoidal) causing blurring of the focusing beam size, Δ*s*_ellip-sphe_, and may degrade energy resolution. However, this is only problematic when the solid angle is much larger and magnification is much smaller. This contribution is neglegible as far as geometries in this article are considered.^3^ ^4^

As a final comment, we note that the off-Rowland case may also be applied without the detector in the analyzer focus. This may be advantageous in some cases.

## 4. Ray-tracing   

Ray-tracing simulations were performed to confirm the accuracy of the analytic formulae of the previous section. Analyzer crystals were taken to be rectangular with dimensions *D*_*x*_ and *D*_*y*_, and to have either a spheroidal or toroidal curvature. (Note that *x* and *y* refer to the two directions perpendicular to the reference X-ray path, *z*, perpendicular and within the scattering plane.) Also, note that while we carefully considered finite extent transverse to the analyzer scattering plane, it had negligible impact in all cases considered. Analyzers are assumed to be ‘diced’ with, for example, crystallite sizes of 0.6 mm × 0.6 mm on a 0.7 mm pitch. The simulations, using geometrical optics, generally traced more than 200000 rays with those rays spread over more than 400 analyzer crystallites, with >400 rays per crystallite. The selected crystallites were uniformly distributed on the analyzer surface in both *x* and *y* directions transverse to the sample–analyzer axis, as were the rays on each crystallite. Each selected crystallite was assumed to have a deviation in orientation from the ideal (spheroidal or toroidal) surface given by a Gaussian distribution to simulate errors in manufacture. The source point (*i.e.* over the sample) for a given ray was also randomly selected for each ray within a Gaussian distribution of size σ_ss_*x*__, σ_ss_*y*__ to simulate the finite beam size on the sample (or finite penetration into the sample). This allows definition of the exact incident angle of each ray onto a crystallite, and, with specular reflection assumed^5^, then defines the point of intersection in the detector.

Aside from the geometric parameters defining the set-up, the reflection curve (in the form of reflected intensity *versus* energy for a fixed and perfectly defined angle near to backscattering) is also required as an input parameter. The transformation from angular deviation to energy shifts was made using Bragg’s law (without linearization). This input reflectivity curve was usually chosen to agree with that calculated from dynamical diffraction from a thick crystal using the Si(*nnn*) series of reflections as listed in Table 1 ▶. However, when only the geometric contribution to the energy was desired, then a narrow delta-function-like reflectivity curve (width <0.05 meV) was used. After setting the geometry and choosing the input reflectivity curve, the incident energy was scanned assuming that the analyzer temperature was held stable. The resulting distributions are then integrated over individual detector pixels, giving curves of intensity as a function of incident energy for a given detector pixel. This is then convolved with an incident energy distribution appropriate for the monochromator defining the bandwidth onto the sample.

## 5. Parameters, results and discussion for meV resolution   

The parameter space is complex, with many free parameters relating to the desired performance and size of the spectrometer. In this section we focus on parameter sets aimed at achieving high, ∼meV, resolution, with an accepted solid angle in the analyzer of 10 mrad, consistent with taking Δ*Q* ≃ 1 nm^−1^. In the next section, §6, we consider medium resolution.

### 5.1. Analyzer and source parameters   

Experience in fabrication of analyzer crystals with large, 9.8 m, radii of curvature (Miwa, 2002 ▶) leads us to take σ_*x*,*y*_ = 20 µrad as the r.m.s. deviation of the analyzer crystallites in each direction. It is possible that this may increase for smaller radii of curvature, but the effect of such deviation, generally scaling as *R*σ, will be reduced by the smaller radius. The source size, or the illuminated volume of the sample projected normal to the sample–analyzer direction, was chosen to be σ_ss(*x*,*y*)_ = 20 µm (47 µm FWHM), consistent with a focused beam at a typical spectrometer. The solid angle of the analyzer crystal in the vertical was fixed at 10 mrad. This is broadly consistent with present spectrometer design.

### 5.2. Spectrometer and detector parameters   

The space between the sample and the detector, *l*, was set at 200 mm, as being comparable with present-day spectrometers with longer 2θ arms. The clearance between the active edge of the detector and the beam was taken as 2 mm, or *d* = *d*_min_ + 2 mm, as discussed above. The detector pixel size was set at *p* = 0.3 mm. In principle, this might be reduced 0.17 or 0.05 mm, consistent with pixel sizes of various detectors. However, the 0.3 mm value is comparable with the effect of blurring owing to analyzer deviation due to the 20 µrad angular variation. It is also consistent with the thickness of typical silicon pixel detectors, which can be the relevant parameter if such a detector is used at grazing incidence to improve the stopping power. In general, while it is easy to consider reducing the pixel size below 0.3 mm, it must be done with care as, to see some benefit from this, many things must be improved simultaneously. The 0.3 mm chosen here is comfortably matched to the present conditions. The (one-dimensional) temperature gradient of the analyzer crystal is assumed to be given by equation (5) or equation (9).

### 5.3. Representative results: energy–position correlation and energy resolution   

As an example, we discuss the parameter set for *L*_1_ = 3 m, listed in Table 4 [(III) and (VI)] ▶. The spheroid surface of Rowland circle diameter *R*_*x*_ = 3000 mm (horizontally) and *R*_*y*_ = 3000 mm (vertically) was taken for case (III). Meanwhile, for case (VI), a toroidal surface of diameter *R*_*x*_ = 3000 mm and *R*_*y*_ = 2897 mm^5^ ^6^ was taken. The energy–position correlation in the detector in this selected geometry is shown in Fig. 5(*a*) ▶ for the on-Rowland geometry. The chromatic aberration owing to the demagnification contribution in Fig. 5(*a*) is reduced by use of the temperature gradient in Fig. 5(*b*), even though fabrication imperfections have been included in §5.1. As shown in Fig. 5(*d*) ▶, the temperature gradient also drastically reduces the aberration from Fig. 5(*c*) ▶.

**Figure 6:**
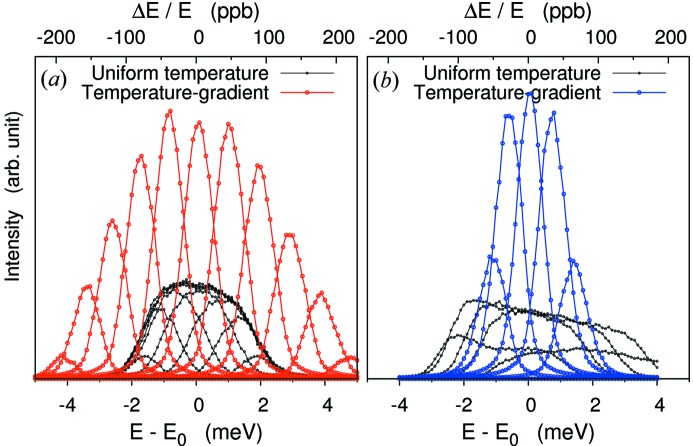
Resolution functions of PSD scanning, incident photon energy 0.1 meV step relative to elastic line. *E* = 21.747 keV for the Si(11 11 11) backreflection case. (*a*) Focus on-Rowland. (*b*) Focus off-Rowland. For comparison, the uniform temperature case of the analyzer is shown. Optical geometry of *L*_1_ = 3 m, *l* = 200 mm, *d* = 4.60 mm (*a*) and *d* = 3.56 mm (*b*) are considered here. Angular deviation from ideal surface σ_(*x*,*y*)_ = 20 µrad × 20 µrad, source size σ_ss(*x*,*y*)_ = 20 µm × 20 µm and intrinsic Darwin width Δ*E*_int_ = 0.8 meV are taken into account. (Incident bandwidth is eliminated.)

Fig. 6 ▶ shows resolution functions from pixels calculated by scanning the incident photon energy across the elastic energy. The FWHM of the spectra of individual pixels gives 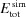 = 1.12 meV^7^ (on-Rowland) and 1.0 meV^8^ (off-Rowland) at Si(11 11 11) assuming a delta-function incident bandwidth. As shown in Fig. 6(*a*) ▶, these agree well with the analytical estimation 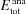 = 1.15 and 1.0 meV (FWHM), respectively. To provide a comparison with a uniform temperature [*T*(*y*_*a*_) = constant], ray-tracing results are also shown for this case in Fig. 6 ▶ (black symbols). In this geometry the energy resolution decreases by a factor of three (on-Rowland) to six (off-Rowland) when the temperature gradient is applied.

### 5.4. Discussion   

**Figure 7:**
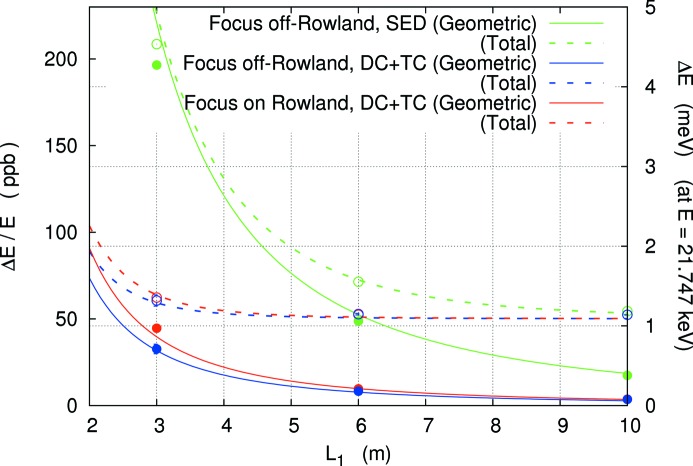
Energy resolution as a function of 2θ arm length *L*_1_ for high-resolution spectrometers. Comparison of a single-element detector (SED) and dispersion compensation (DC) with temperature compensation (TC). Closed and open circles represent simulation results of geometric and total contributions, respectively. Solid lines are the geometric terms discussed in Table 4 ▶. Broken lines are the estimated total resolution including non-perfection contributions.

Here, we consider the dependence of the energy resolution on the spectrometer size, *L*_1_. Using analytic forms discussed in §2, the energy resolution as a function of 2θ arm length *L*_1_ is summarized in Fig. 7 ▶, expressed by solid (geometric contribution) and broken (total contribution) lines.^9^ Table 4 ▶ lists the results of Fig. 7 ▶. Ray-tracing results, using parameters in Table 4 ▶, are shown by circles. This shows that it is possible to estimate resolution using the analytic approximations with a fair degree of accuracy. The effect of the temperature gradient becomes large beginning near 6 m. An energy resolution of ∼1.5 meV is possible at 21.7 keV for *L*_1_ > 3 m.

## 6. Medium-resolution with large angular acceptance   

We now consider application to medium-resolution large-solid-angle analyzers. This is the case originally considered for dispersion compensation without a temperature gradient (Huotari *et al.*, 2005 ▶, 2006 ▶). While more space is available near the sample in this case since the resolution is relaxed, it is still limited, so it is attractive to consider moving the detector away from the sample. In contrast to high-resolution IXS, medium-resolution set-ups often employ large-solid-angle analyzers (Ω = 50–100 mrad) to increase count rate. In this case, while the formulae given in §3 remain applicable as a first approximation, some care is needed and ray-tracing becomes increasingly important. Here we focus on shorter (1–2 m-long) arms and a fixed large analyzer crystal *D*_(*x*,*y*)_ = 100 mm. We consider the Si(555) reflection at *E* = 9.9 keV which has an intrinsic resolution (single reflection) of 14.6 meV. We take *l* = 100 mm.

**Figure 8:**
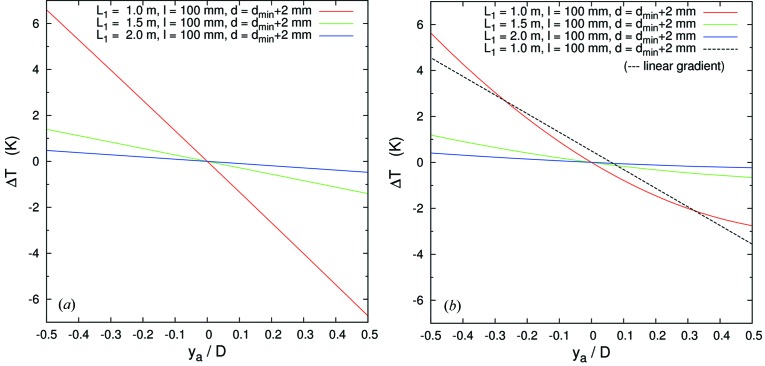
Temperature gradient for medium resolution for (*a*) focus on-Rowland [case (III)] and (*b*) focus off-Rowland [case (VI)]. The temperature deviation Δ*T* as a function of analyzer *y*-position (*y*_a_) normalized by analyzer dimension *D* = 100 mm is shown for silicon analyzers with central temperature 300 K.

**Figure 9:**
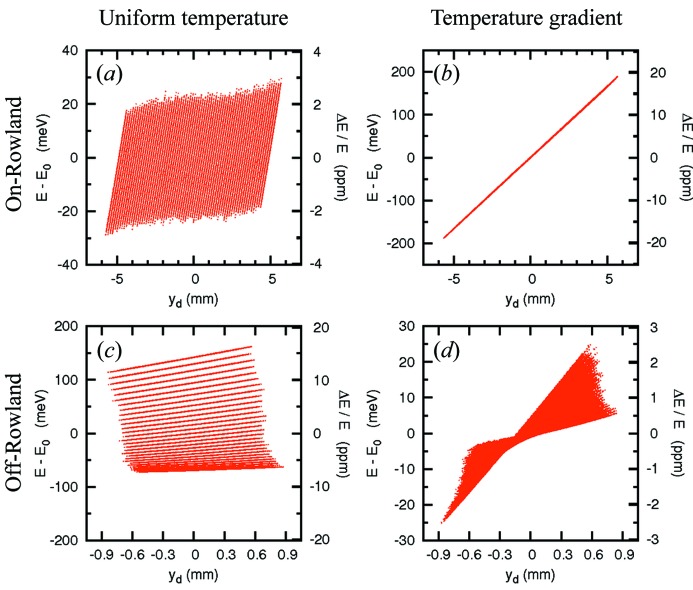
Energy–position correlation in the detector by ray-tracing. *E* = 9.885 keV, Si(555) backreflection case. *y*_d_: detector vertical position. *E* − *E*_0_: relative energy. Optical geometry of *L*_1_ = 1 m, *l* = 100 mm and (*a*) *d* = 12.60 mm and (*b*) *d* = 7.54 mm are considered here. Contributions from slope error σ_(*x*,*y*)_ = 20 µrad × 20 µrad, source size σ_ss(*x*,*y*)_ = 20 µm × 20 µm are included

**Figure 10:**
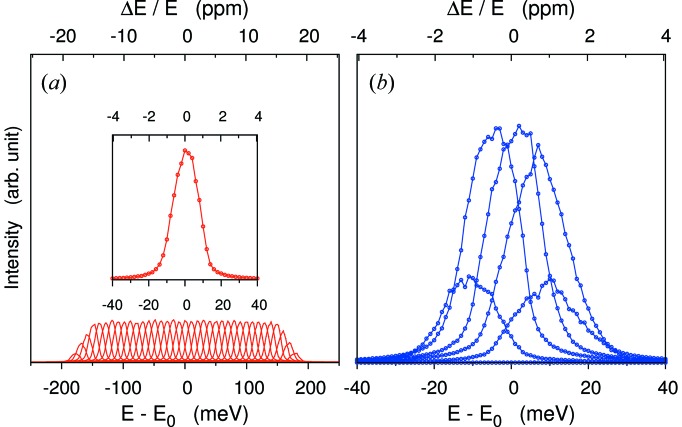
Resolution functions: (*a*) focus on-Rowland (*b*) focus off-Rowland. The inset in (*a*) is magnification of results for one pixel. *E* = 9.885 keV at Si(555) backreflection. *L*_1_ = 1 m, *l* = 100 mm, *p* = 0.3 mm, (*a*) *d* = 12.6 mm and (*b*) *d* = 7.54 mm. The angular deviation from ideal surface σ_(*x*,*y*)_ = 20 µrad × 20 µrad, source size σ_ss(*x*,*y*)_ = 20 µm × 20 µm and intrinsic Darwin width Δ*E*_int_ = 14.7 meV (FWHM) are taken into account. (Incident bandwidth is not included.)

In contrast to the high-resolution analyzers, the temperature gradient of the present case (smaller arm and large solid angle) becomes much steeper as seen in Fig. 8 ▶. The corresponding energy–position characteristics are shown in Fig. 9 ▶. Another important point is that the magnitude of the image at the detector increases quickly with increasing Ω*l*. When Ω = 100 mrad, *l* = 100 mm, one can estimate the image size to be 11.2 mm. This is much larger than one-to-one focusing (on-Rowland geometry) image size (2*c* = 1.2 mm) and requires a large number of detector pixels (see Fig. 10*a* ▶), and may make the off-Rowland geometry relatively attractive.

**Figure 11:**
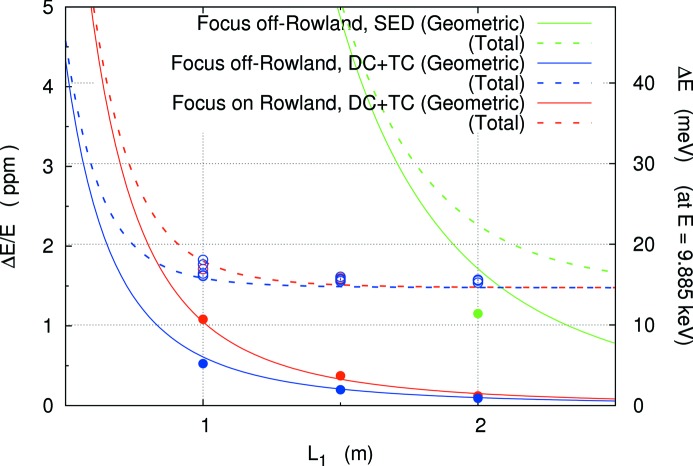
Energy resolution as a function of 2θ arm length, *L*_1_, for medium-resolution spectrometers. Comparison of a single-element detector (SED) and dispersion compensation (DC) with temperature compensation (TC). Closed and open circles represent simulation results of geometric and total contributions, respectively. Solid lines are geometric terms discussed in Table 5 ▶.

The energy resolution as a function of *L*_1_ is shown in Fig. 11 ▶ ▶. One can obtain a resolution almost the same as the intrinsic reflection width Δ*E* ≃ 15 meV listed in Table 1 ▶. This drastically increases when *L*_1_ < ∼1 m. For Δ*E* < 20 meV, one requires *L*_1_ > ∼1 m.

**Figure 12:**
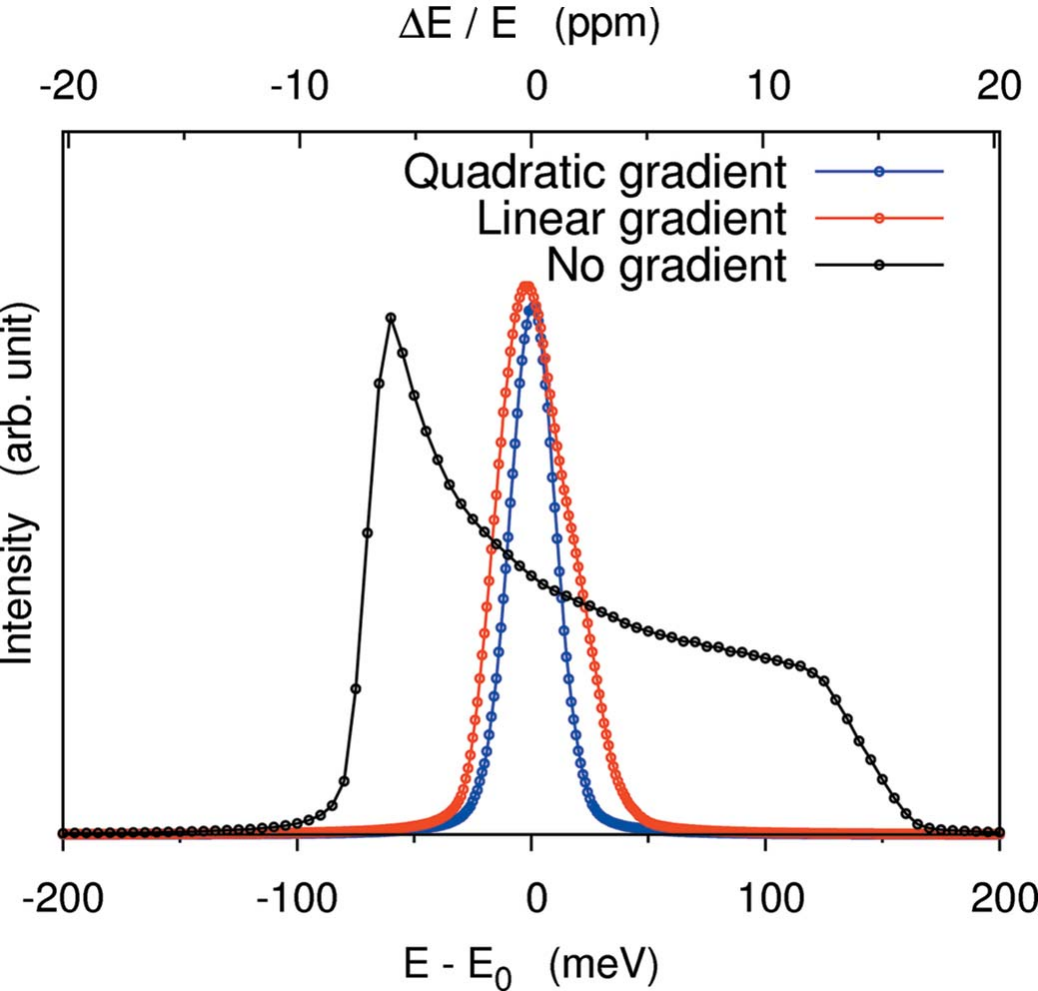
Improved energy resolution using a single-element detector and a temperature gradient in the off-Rowland geometry. Conditions for the simulations are large solid angle, 100 mrad, *E* = 9.885 keV of Si(555) with three different temperature gradients. See text for discussion.

Before closing this section, it is worth noting that the application of a temperature gradient can improve the energy resolution even when a single-element detector is used. This works in the off-Rowland geometry [case (VI) in Fig. 2 ▶]. Fig. 12 ▶ shows results for *L*_1_ = 1 m, Ω = 100 mrad, *l* = 100 mm. Ray-tracing results are shown for quadratic temperature gradient Δ*E* = 23 meV (FWHM) and a more practical linear gradient Δ*E* = 35 meV (FWHM). These are much better than without the gradient which has an asymmetric line shape with Δ*E* = 72 meV (FWHM) or Δ*E* = 232 meV (full width at tenth of maximum). Similar improvements, though not as dramatic, are also possible in high-resolution configurations.

## 7. Some practical considerations   

The practical aspects of detector size, momentum-resolution and noise are mentioned in this section. While from the point of view of the dispersion and energy resolution the on-Rowland case is preferable, it leads to a relatively large beam size at the detector, so requires a larger detector and larger *d*_min_. Background in the detector is usually dominated by cosmic-ray muon events, and can be expected to scale with area, so the on-Rowland case will have a larger noise, and one should consider count rates in expected experiments carefully. The increased offset, larger *d*_min_, may also become more of an issue as one considers a two-dimensional analyzer array (Baron *et al.*, 2008 ▶).

The on-Rowland case, however, offers the possibility to improve momentum resolution using transverse position sensitivity of the detector. The essential idea is that if the detector is not in the horizontal analyzer focus then there is a correlation between horizontal detector position and horizontal analyzer position. In particular, assuming a spherical analyzer the beam size for the on-Rowland case is just Ω*l* while the blurring owing to the crystallite size (pinhole effect) is just 2*c*′. Then, if Ω*l* 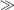 2*c*′, the detector position sensitivity allows one effective momentum resolution. A correlatory to this is that if one could obtain a single analyzer crystal with very large extent out of the scattering plane, then the position sensitivity might be sufficient such that the single crystal would act as an array. Thus a horizontal analyzer array might be avoided. However, as the limit for analyzer fabrication, at least for high resolution, is really the dicing and bonding process, this would require significant advances in analyzer fabrication technique. It would probably be most interesting for shorter radius arm, where, for example, one might consider a toroidal analyzer, with different radii in the vertical and horizontal, so that the vertical radius might be chosen to match the off-Rowland conditions and so reduce the detector extent, while the horizontal might be chosen to allow the momentum resolution to be determined by the detector.

## 8. Preliminary temperature gradient experiment   

**Figure 13:**
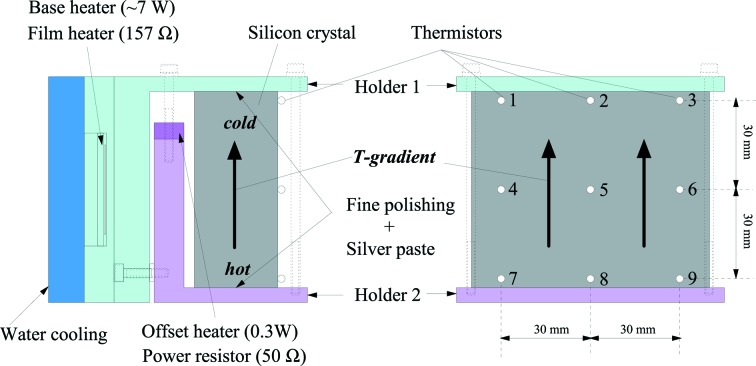
Sketch of a temperature gradient crystal holder. Numbers on the right-hand figure represent the serial numbers of thermistors and are referred to in Table 6 ▶. Crystal surface mount thermistors and monitored temperature at each position.

We tested one possibility for creating the required temperature gradient. Fig. 13 ▶ shows a schematic of our apparatus. A rectangular piece of silicon is used to simulate the analyzer substrate, and is placed between two copper plates. The silicon analyzer can then be considered as one element in a thermal circuit: passing a constant heat flow through the silicon should create the desired gradient. Considering the thermal conductivity of silicon, 1.3 W cm^−1^ K^−1^ at room temperature, and choosing the silicon cross section to be 3 cm × 9 cm (normal to the flow), one expects that a heat flow of ∼0.5 W will create a temperature difference of 100 mK across 7 cm of silicon.

To test this, we place the holder sketched in Fig. 13 ▶ into a vacuum. The base temperature was controlled by a PID system and the offset heater was held at a constant power. The total power to the base heater was about 7 W, while the offset heater was 0.3 W. The temperature distribution over the surface was measured by nine calibrated thermistors that were attached to the surface using silver paste. As one can see from the results in Table 6 ▶, the gradient was controllable to within ±3 mK, along a horizontal line. This level of control should allow reduction of a geometrically broadened resolution of 2.2 meV to about 0.6 meV at 22 keV, a reasonable first starting point for this work.

## 9. Conclusions   

The promise of inelastic X-ray scattering has always been offset by the complexity of the necessary spectrometers. However, increased experience, improvements in optics, detectors and overall beamline design make it increasingly possible to consider very sophisticated instrumentation. In contrast, hutch size, and space on the experimental floor remain serious limitations. Thus the suggestion of introducing a temperature gradient on analyzer crystals to reduce spectrometer size for a given resolution, or improve resolution for a fixed size, is both timely and relevant.

Our work suggests ∼1.5 meV energy resolution should be possible at 21.7 keV using a 3 m arm while keeping 200 mm clearance between the sample and the detector, and better than 20 meV resolution at 10 keV with 100 mm clearance. Other points discussed include the possibility to improve the energy resolution even with single-element detectors when the analyzer focus is not on the Rowland circle, and the possibility of using a two-dimensional position-sensitive detector for improving momentum resolution transverse to the analyzer scattering plane without slitting.

## Figures and Tables

**Table 1:** Properties of the Si(*nnn*) series in an almost backscattering geometry. *E*_1Flat_: calculated intrinsic single reflection bandwidth; *E*_2Flat_: measured bandwidth from two flat crystals (Baron *et al.*, 2000 ▶); *E*_Ana_: typical observed total energy resolution with an analyzer crystal. Parentheses indicate calculated values

*n*	*E* (keV)	*E*_1Flat_ (meV)	*E*_2Flat_ (meV)	*E*_Ana_ (meV)
5	9.885	(14.5)	(21.0)	
7	13.839	(4.8)	(6.9)	
8	15.816	(4.1)	(5.8)	6.0
9	17.793	(1.8)	2.4	3.0
11	21.747	(0.8)	1.2	1.5
12	23.725	(0.75)	1.1	
13	25.702	(0.35)	0.6	0.9

**Table 2:** Definition of parameters See also Figs. 1 ▶ and 2 ▶. In some cases subscripts *x* and *y* are used to indicate horizontal (out of the analyzer scattering plane) and vertical (in the analyzer scattering plane).

= *E*/*E*	Fractional energy resolution
_1_	Geometric contribution to the resolution in a conventional configuration
_2_	Geometric contribution to the resolution when a position-sensitive detector is used with the Rowland-circle condition satisfied; applies to both the case when the detector is in the analyzer focus (dispersion compensation) or out of the focus (with temperature gradient)
_3_	Demagnificaton contribution to the resolution when the Rowland-circle condition is violated without a temperature gradient
_4_	Contribution to the resolution when the Rowland-circle condition is violated with a temperature gradient
= /2 _B_	Deviation from exact backscattering
_0_	Deviation from backscattering at the center of the analyzer crystal
*R*	Radius of curvature of the analyzer crystal
*L*_1_	Distance from sample to analyzer crystal
*L*_2_	Distance from analyzer crystal to analyzer focal point
	Note: always have the thin lens equation 2/*R* = 1/*L*_1_ + 1/*L*_2_
	Note: on-Rowland is the case *R* = *L*_1_ = *L*_2_
*d*	Detector offset transverse to the beam path from center of sample to center of detector
*l*	Shift of the detector away from the sample toward the analyzer crystal
*p*	Detector pixel size transverse to beam direction in scattering plane
*c*	Crystallite transverse dimension
*D*	Size of the analyzer crystal in the scattering plane
*M*	Magnification *L*_2_/*L*_1_
_min_	Deviation on line from center of sample to lower edge of analyzer to center of detector _min_ (1/4){2*d*/*R* [(1 *M*)/*M*]}
_max_	Deviation on line from center of sample to upper edge of analyzer to center of detector _min_ (1/4){2*d*/*R* + [(1 *M*)/*M*]}
	Distributions of the angles onto the analyzer
	_max_ _min_ = (/2)[(1 *M*)/*M*]
	Note: upward scattering so that _max__0__min_
*y*_a_	Vertical position from center of analyzer
*y*_d_	Vertical position from center of detector
*d*_min_	Minimum detector offset of *d*
*T*_0_	Temperature at center of analyzer
*T*_min_	Minimum temperature of analyzer
*T*_max_	Maximum temperature of analyzer
*T*	Difference of temperature from center of analyzer ( *T* *T*_0_)
*E*	Difference of energy of rays from elastic energy ( *E* *E*_0_)
2*c*	Demagnified vertical image size by off-Rowland geometry [ *c*(1 + *M*)]

**Table 3:** Properties of two focusing geometries: focus on-Rowland circle [case (III)] and focus off-Rowland circle [case (VI)] Notation of the optical parameters are explained in the text.

	Focus on-Rowland	Focus off-Rowland
Sampledetector minimum vertical offset, *d*_min_	*l* + *c* (larger)	*l*/2 + *c* (smaller)
Image size (detector active area, *y*-direction)	*l* + 2*c* (larger)	2*c* (smaller)
Energyposition correlation	Linear	Quadratic
Temperature gradient	Linear	Quadratic
Energy resolution	_2_ = (*p*/2*R*)tan_0_	_4_ = (*p*/2*R*)tan_min_ + (*c*/8*R*)

**Table 4:** Calculated contributions to energy resolution in (I)(VI) Parameters are defined in Table 2 ▶. Analyzer crystal dimension *D* = 100, 60 and 30mm is chosen to keep the solid angle = 10mrad. *T*_1_ and *T*_2_ are top and bottom temperature offset relative to the analyzer center. _1_ to _4_ are contributions to the energy resolution discussed in the text. (*E*)_geom_: geometric energy resolution at *E* = 21.747keV. (*E*)_sim_: ray-tracing results, only geometric contributions are taken into account. Si(13 13 13) backreflection case at *E* = 25.702keV are also shown in (*E*)_geom_.

	(I)(*a*)			(II)(*a*)			(III)			(IV)			(V)			(VI)		
*L*_1_ (m)	10	6	3	10	6	3	10	6	3	10	6	3	10	6	3	10	6	3
*L*_2_ m)	10	6	3	10	6	3	10	6	3	9.8	5.8	2.8	9.8	5.8	2.8	9.8	5.8	2.8
*R* (m)	10.00	6.000	3.000	10.00	6.000	3.000	10.00	6.000	3.000	9.899	5.898	2.897	9.899	5.898	2.897	9.899	5.898	2.897
*c* (mm)	0.6	0.6	0.6	0.6	0.6	0.6	0.6	0.6	0.6	0.6	0.6	0.6	0.6	0.6	0.6	0.6	0.6	0.6
*d* (mm)	2.60	2.60	2.60	2.60	2.60	2.60	4.60	4.60	4.60	3.59	3.59	3.58	3.59	3.59	3.58	3.59	3.59	3.58
*l* (mm)	0.0	0.0	0.0	0.0	0.0	0.0	200	200	200	200	200	200	200	200	200	200	200	200
*p* (mm)				0.3	0.3	0.3	0.3	0.3	0.3				0.3	0.3	0.3	0.3	0.3	0.3
*M*	1.00	1.00	1.00	1.00	1.00	1.00	1.00	1.00	1.00	0.98	0.97	0.93	0.98	0.97	0.93	0.98	0.97	0.93
_0_ (mrad)	0.13	0.22	0.43	0.13	0.22	0.43	0.23	0.39	0.79	0.18	0.30	0.62	0.18	0.30	0.62	0.18	0.30	0.62
(mrad)	0.0	0.0	0.0	0.0	0.0	0.0	0.0	0.0	0.0	0.10	0.17	0.36	0.10	0.17	0.36	0.10	0.17	0.36
*T*_1_ (mK)	0	0	0	0	0	0	4.5	12.6	52.1	0	0	0	0	0	0	3.0	8.5	35.7
*T*_2_ (mK)	0	0	0	0	0	0	4.5	12.6	52.1	0	0	0	0	0	0	4.0	11.4	47.8
_1_ (p.p.b.)	8	22	87															
_2_ (p.p.b.)				2	5	22	3	10	40									
_3_ (p.p.b.)										19	52	221	19	52	221			
_4_ (p.p.b.)																3	8	32
(p.p.b.)	8	22	87	2	5	22	3	10	40	19	52	221	19	52	221	3	8	32
(*E*)_geom_ (meV)†	0.17	0.47	1.88	0.04	0.12	0.47	0.08	0.21	0.86	0.40	1.14	4.80	0.40	1.14	4.80	0.06	0.17	0.69
(*E*)_sim_ (meV)	0.16	0.46	1.86	0.06 (0.002)	0.12 (0.01)	0.47 (0.06)	0.08	0.21	0.97	0.38	1.06	4.27	0.40 (0.03)	1.11 (0.10)	4.4 (0.4)	0.0680.078	0.150.18	0.590.71
(*E*)_geom_ (meV)‡	0.20	0.56	2.23	0.05	0.14	0.56	0.09	0.25	1.02	0.48	1.34	5.64	0.48	1.34	5.64	0.07	0.20	0.81

† *E* = 21.747keV.

‡ *E* = 25.702keV.

**Table 5:** Calculated contributions to the energy resolution for medium-resolution spectrometers operating at the Si(5 5 5) reflection (short arm length and large solid angle) *L*_1_ = 2.0, 1.5 and 1.0m and *c* = 0.6mm, *l* = 100mm, *p* = 0.3mm and *D* = 100mm (corresponding solid angles are = 100, 66.7 and 50mrad) are selected. We take *d*= 10mm for cases (I) and (II) and *d* = *d*_min_ + 2mm for cases (III)(VI). (*E*)_geom_: geometric energy resolution at *E* = 9.885keV. Results for Si(7 7 7) reflection case at *E* = 13.839keV are shown in the last row.

	(I)(*a*)			(II)(*a*)			(III)			(IV)			(V)			(VI)		
*L*_1_ (m)	2.0	1.5	1.0	2.0	1.5	1.0	2.0	1.5	1.0	2.0	1.5	1.0	2.0	1.5	1.0	2.0	1.5	1.0
*L*_2_ (m)	2.0	1.5	1.0	2.0	1.5	1.0	2.0	1.5	1.0	1.9	1.4	0.9	1.9	1.4	0.9	1.9	1.4	0.9
*R* (m)	2.0	1.5	1.0	2.0	1.5	1.0	2.0	1.5	1.0	1.949	1.448	0.947	1.949	1.448	0.947	1.949	1.448	0.947
*c* (mm)	0.6	0.6	0.6	0.6	0.6	0.6	0.6	0.6	0.6	0.6	0.6	0.6	0.6	0.6	0.6	0.6	0.6	0.6
*d* (mm)	10.0	10.0	10.0	10.0	10.0	10.0	7.60	9.27	12.6	5.09	5.91	7.57	5.09	5.91	7.57	5.09	5.91	7.57
*l* (mm)	0.0	0.0	0.0	0.0	0.0	0.0	100	100	100	100	100	100	100	100	100	100	100	100
*p* (mm)				0.3	0.3	0.3	0.3	0.3	0.3				0.3	0.3	0.3	0.3	0.3	0.3
*M*	1.0	1.0	1.0	1.0	1.0	1.0	1.0	1.0	1.0	0.95	0.93	0.90	0.95	0.93	0.90	0.95	0.93	0.90
_0_ (mrad)	2.50	3.33	5.00	2.50	3.33	5.00	1.95	3.20	6.63	1.30	2.04	3.98	1.30	2.04	3.98	1.30	2.04	3.98
(mrad)	0.0	0.0	0.0	0.0	0.0	0.0	1.3	2.4	5.6	1.3	2.4	5.5	1.3	2.4	5.5	1.3	2.4	5.5
*T*_1_ (K)	0.0	0.0	0.0	0.0	0.0	0.0	0.48	1.40	6.70	0.0	0.0	0.0	0.0	0.0	0.0	0.24	0.65	2.74
*T*_2_ (K)	0.0	0.0	0.0	0.0	0.0	0.0	0.48	1.40	6.70	0.0	0.0	0.0	0.0	0.0	0.0	0.41	1.19	5.67
_1_ (p.p.m.)	0.75	1.33	3.00															
_2_ (p.p.m.)				0.19	0.33	0.75	0.15	0.32	0.99									
_3_ (p.p.m.)										1.72	4.86	22.2	1.72	4.86	22.2			
_4_ (p.p.m.)																0.10	0.21	0.61
(p.p.m.)	0.75	1.33	3.00	0.19	0.33	0.75	0.15	0.32	0.99	1.72	4.86	22.2	1.72	4.86	22.2	0.10	0.21	0.61
(*E*)_geom_ (meV)†	7.4	13.2	29.7	1.9	3.3	7.4	1.4	3.2	9.8	17.0	48.0	219	17.0	48.0	219	0.98	2.04	6.01
(*E*)_sim_ (meV)	7.4	13.1	29.6	1.8	3.3	7.2	1.2	3.7	10.7	11.4‡	22.5 (48)‡	57.7‡	5.4, 4.9, 2.9‡	11, 17, 13 (48)‡	53, 78, 40 (57)‡	0.90	1.97	5.2
(*E*)_geom_ (meV)§	11.9	21.1	47.4	3.0	5.3	11.9	2.0	4.4	13.8	23.7	67.0	306.0	23.7	67.0	306.0	2.7	6.6	23.1

† *E* = 9.885keV.

‡ The differences between the analytic estimation and the simulations are due to asymmetric resolution function from the non-linear energyposition correlations.

§ *E* = 13.839keV.

**Table 6:** Example of a temperature gradient result

(1) 27.338	(2) 27.341	(3) 27.337
(4) 27.359	(5) 27.359	(6) 27.360
(7) 27.381	(8) 27.379	(9) 27.381

## Footnotes

1 In fact, nuclear resonant scattering (the Mössbauer effect) offers alternative methods of high-resolution analysis, either with the resonant isotope embedded in the sample (Seto *et al.*, 1995 ▶) or as an external analyzer foil (Chumakov *et al.*, 1996 ▶). However, the former is limited to samples containing the resonant isotope and only gives density of states information (being essentially an absorption measurement) while the latter is hampered by the mismatch of nuclear analyzer bandwidth (typically microvolts, or less) and the ∼meV monochromator bandwidth.

2 To obtain sub-meV resolution, we consider backreflection of Si(13 13 13), which gives Δ*E* = 0.3 meV at *E* = 26 keV.

3 Strictly speaking, 2/(*R*cosδ_0_) = 1/*L*_1_ + 1/*L*_2_ is the best focusing condition. However, in this paper, δ_0_ is small so that one can omit the cosδ_0_ term: δ_0_ is 10 mrad then 1 − cosδ_0_ is only 5 × 10^−5^.

4 The maximum spherical aberration is (*D* ^3^/16*R* ^2^)[(1 − *M* ^2^)/*M* ^2^]. One can calculate a worst-case blurring of ∼16 µm for *L*_1_ = 1 m, *l* = 0.1 m and Ω = 100 mrad.

5 Effects from the finite penetration into the analyzer crystal (which can be estimated to spread a well defined beam, over a length ∼2δ_0_λ_abs_) were neglected. This contribution is much smaller than cube size or detector pixel size so that one can assume reflection occurs specularly at the surface of the flat cube.

6 The horizontal radius of curvature *R*_*x*_ affects the horizontal size of the focused beam but does not affect the energy resolution or the temperature gradient for cases considered in this paper. See also §7.

7 The geometric term of the resolution is calculated as  = 0.97 meV (FWHM)

8
 = 0.59–0.71 meV (FWHM) depending on the position of the detector pixels.

9 ∊_tot_ ≡ . Here, ∊_slope_ and ∊_source_ are given by 2.35σtanδ_0_ and 2.35σ_ss_tanδ_0_, respectively. ∊_int_ is the intrinsic reflection width of a specified diffraction plane.
